# *Glaesserella parasuis* autotransporters EspP1 and EspP2 are novel IgA-specific proteases

**DOI:** 10.3389/fmicb.2022.1041774

**Published:** 2022-12-15

**Authors:** Zhichao Wang, Jiayun Gu, Kunxue Xiao, Wenlong Zhu, Yan Lin, Siting Wen, Qigai He, Xiaojuan Xu, Xuwang Cai

**Affiliations:** ^1^State Key Laboratory of Agricultural Microbiology, College of Veterinary Medicine, Huazhong Agricultural University, Wuhan, China; ^2^Key Laboratory of Preventive Veterinary Medicine in Hubei Province, The Cooperative Innovation Center for Sustainable Pig Production, Wuhan, China

**Keywords:** *Glaesserella parasuis*, EspP1, EspP2, IgA-protease, immune evasion

## Abstract

**Background:**

*Glaesserella parasuis* causes Glässer’s disease, which is associated with severe polyarthritis, fibrinous polyserositis and meningitis, and leads to significant economic losses to the swine industry worldwide. IgA is one of the most important humoral immune factors present on mucosal surfaces, and it plays a crucial role in neutralizing and removing pathogens. *G. parasuis* is able to colonize the mucosal membrane of respiratory tract without being eliminated. Nevertheless, the immune evasion mechanism of *G. parasuis* in thwarting IgA remains unclear.

**Aims:**

The object of this study is to characterize the IgA degradation activity of Mac-1-containing autotransporter EspP1 and EspP2 from *G. parasuis*.

**Methods:**

The swine IgA was purified and incubated with EspP1 and EspP2 respectively. Western blotting was used to detect the cleavage of swine IgA. Generation of EspP1 and EspP2 mutant protein were used to explore the putative active sites of EspPs. LC-MS/MS based N/C-terminal sequencing was performed to measure the cleavage sites in swine IgA.

**Result:**

Our results show that *G. parasuis* EspP1 and EspP2 cleave swine IgA in a dose- and time- dependent manner. *G. parasuis* lose the IgA protease activity after simultaneously delete *espP1* and *espP2* indicating that EspP1 and EspP2 are the only two IgA proteases in *G. parasuis*. The IgA protease activity of EspP1 and EspP2 is affected by the putative active sites which contain Cys47, His172 and Asp194/195. Swine IgA is cleaved within Cα1 and Cα3 domains upon incubation with EspPs. Moreover, EspPs can degrade neither IgG nor IgM while *G. parasuis* possess the ability to degrade IgM unexpectedly. It suggests that *G. parasuis* can secrete other proteases to cleave IgM which have never been reported.

**Conclusion:**

We report for the first time that both EspP1 and EspP2 are novel IgA-specific proteases and cleave swine IgA within the Cα1 and Cα3 domains. These findings provide a theoretical basis for the EspPs-induced immune evasion.

## Introduction

*Glaesserella parasuis* is the causative agent of Glässer’s disease, which causes significant economic losses to the swine industry (Cerda-Cuellar et al., 2010; Zhang et al., 2014). *G. parasuis* belongs to opportunistic bacteria and it is an early colonizer of the upper respiratory tract of piglets and is able to invade host and cause Glässer’s disease with high morbidity and mortality under certain conditions. *G. parasuis* is frequently isolated from the upper respiratory tract of healthy pigs (Cerda-Cuellar et al., 2010; Macedo et al., 2014), suggesting that it is able to escape the elimination and clearance from the host immune system, however, the mechanism remains unclear.

Mammalian respiratory system consists of the upper respiratory tract and the lower respiratory tract. As the entrance of the ambient air, the upper respiratory tract is exposed to a variety of microbes that can cause inflammatory response once colonizing the lower respiratory tract, and it must defend against invasion of the pathogens (Murphy et al., 2009). Immunoglobulin A is essential to the process that mucosal immunity mediates elimination and clearance of the pathogens (de Sousa-Pereira and Woof, 2019). Secretory IgA (sIgA) is the major immunoglobulin in mucosal secretions which is mostly in dimeric or polymeric form of serum-type IgA (Kurono, 2022). The sIgA of mucosal surface directly engages with antigens on pathogens through its antigen binding sites to prevent attachment to host cells from pathogens, and the Fab regions of IgA are responsible for binding to antigen, linked to Fc region *via* hinge region. Afterwards, Fc region interacts with host receptor, known as FcαRI, to trigger elimination processes (de Sousa-Pereira and Woof, 2019). Many pathogenic bacteria exhibit IgA protease activity that include but are not limited to *Haemophilus influenzae*, *Streptococcus pneumoniae*, and *Mannheimia haemolytica* (Clementi et al., 2014; Janoff et al., 2014; Ayalew et al., 2019). Previous research has shown that *G. parasuis* also exists the IgA protease activity to cleave the swine IgA heavy chain, but no genes were identified in *G. parasuis* genome that homology to the *H. influenzae* IgA protease genes *iga* and *igaB* (Mullins et al., 2011).

A previous study of our group has shown that 24 genes of *G. parasuis* are uniquely expressed during bacterial infection by *in vivo*-induced antigen technology (IVIAT), and the autotransporter EspPs belong to one of them (Mao et al., 2020). It suggests that EspP1 and EspP2 may be of great importance in natural infection. The type V-secreted serine protease EspP of *Escherichia coli* has been reported to have proteolytic activity for several substrates such as pepsin A, factor V (Brunder et al., 1997), complement factors C3/C3b and C5 (Orth et al., 2010), and it influences adherence of *E. coli* O157:H7 to bovine primary rectal epithelial cells (Dziva et al., 2007). Yet, *G. parasuis* EspPs show less conservation among EspPs of other bacteria. The result of protein analysis *via* Pfam database^1^ shows that both EspP1 and EspP2 contain a Mac-1 like domain. Mac-1, also known as IdeS, is capable of cleaving immunoglobulin. IdeS of *Streptococcus pyogenes* is an endopeptidase with specificity for IgG (von Pawel-Rammingen et al., 2002), while IdeS of *Streptococcus suis* is an IgM-specific protease (Seele et al., 2013). The EspP1 and EspP2 may be important virulence factors of *G. parasuis.*

## Materials and methods

### Bacterial strains, plasmids, and growth conditions

The bacterial strains and plasmids used in the present study are listed in Table 1. The virulent serovar 5 *G. parasuis* CF7066 was cultivated on tryptic soy agar (TSA) or in tryptic soy broth (TSB; Difco Labotatories, Detroit, MI, United States) supplemented with 5% bovine serum and 10 μg/ml nicotinamide adenine dinucleotide (NAD) at 37°C. *Escherichia coli* DH5α and BL21 (DE3) were grown in Luria-Bertani (LB) medium at 37°C. Agar (1.5%) was included when solid medium was desired. For selection and maintenance of the plasmid-containing strains, the culture medium was supplemented with 50 μg/ml kanamycin (Biofroxx, Darmstadt, Germany).

**Table 1:** Bacterial strains and plasmids used in this study.

Strains or plasmids	Relevant characteristic	Source
*G. parasuis* strains		
CF7066	Serovar 5, wild type strain	Laboratory collection
CF7066Δ*espP1*	*espP1* gene is replaced with an erythromycin resistance cassette	Laboratory collection
CF7066Δ*espP2*	*espP2* gene is replaced with a kanamycin resistance cassette	Laboratory collection
CF7066Δ*espP1*Δ*espP2*	*espP1* and *espP2* are deleted simultaneously and replaced with erythromycin and kanamycin resistance cassette	Laboratory collection
*E. coli* strains		
DH5α	Standard cloning vector	Invitrogen, Carlsbad, CA, USA
BL21 (DE3)	Standard expression vector	Invitrogen, Carlsbad, CA, USA
Plasmids		
pET-28a	An expression vector, containing N/C-terminal His-tag, Kan^r^	Novagen, Madison, WI, USA
pET-28a-*espP1*	pET-28a containing *espP1* wild type	This study
pET-28a-*espP1*^C47A^	pET-28a containing *espP1* ^C47A^	This study
pET-28a-*espP1*^D144A^	pET-28a containing *espP1* ^D144A^	This study
pET-28a-*espP1*^H172A^	pET-28a containing *espP1* ^H172A^	This study
pET-28a-*espP1*^D195A^	pET-28a containing *espP1* ^D195A^	This study
pET-28a-*espP2*	pET-28a containing *espP2* wild type	This study
pET-28a-*espP2*^C47A^	pET-28a containing *espP2* ^C47A^	This study
pET-28a-*espP2*^D144A^	pET-28a containing *espP2* ^D144A^	This study
pET-28a-*espP2*^H172A^	pET-28a containing *espP2* ^H172A^	This study
pET-28a-*espP2*^D194A^	pET-28a containing *espP2* ^D194A^	This study

Kan^r^, kanamycin-resistance.

### Purification of swine IgA

Purification of swine IgA was adapted from a published protocol (Bourne, 1969) with slight modifications. Briefly, secretory IgA was purified from fresh swine colostrum. After adding 50% saturated ammonium sulfate in whey to precipitate immunoglobulins, pellets were collected by centrifugation at 200 × *g* and resuspended in PBS. Ammonium sulfate was removed by dialysis in PBS for 3 days. Swine IgA was purified on Sephadex G-200 (SolarBio Life Sciences, Beijing, China) and eluted with PBS. Fraction from the first peek was pooled and concentrated. The concentrated fraction was then applied to a column of DEAE-52 (SolarBio Life Sciences, Beijing, China) which was eluted by the following stepwise changes of molarity of NaCl at the same pH: 0.1, 0.3, and 1.0 M. IgA was eluted at 0.3 M NaCl.

### Cloning, expression, purification of recombinant protein and its mutant proteins

All recombinant proteins encoding sequences were amplified from genomic DNA of *G. parasuis* strain CF7066. The primers used in this study are shown in Table 2. *G. parasuis* genes *espP1* and *espP2* were inserted, respectively, into pET-28a using *Bam*HI, *Xho*I, and T4 DNA Ligase (New England Biolabs, Ipswich, MA, United States). The plasmids containing *G. parasuis* genes were individually transformed into *E. coli* DH5α or BL21 to express His-EspP1/His-EspP2 fusion protein.

**Table 2:** Primers used in this study.

Primers	Sequences (5′–3′)	Size (bp)
espP1-F/R	GCGGGATCCGACGATGTCTACTGGG	2,766
	CCGCTCGAGGAACGAGTATCTTACATTGG	
espP1^C47A^-F/R	TATCCTAACCAAGCCTGGGGAGCTGTTGCAGGA	8,101
	CCAGGCTTGGTTAGGATATTGAAAATCAGCAGT	
espP1^D144A^-F/R	GCGTTACGCCTCTAATGCAGCTTTAGTTACAAAATCAT	8,100
	CATTAGAGGCGTAACGCTCAGTCCAAAATGGAC	
espP1^H172A^-F/R	ATGGACAGCCACTGTGACTTTATGGGGCATTGA	8,100
	TCACAGTGGCTGTCCATGATGTTAGCGCGGCA	
espP1^D195A^-F/R	TATCAGTGCCTCTGTAGCAGATCAGGCAGGAA	8,100
	CTACAGAGGCACTGATATAACCTTTTTTAATTTTGCC	
espP2-F/R	CGCGGATCCCAGACTTATTGGGCAAG	2,289
	CCGCTCGAGGAACGAGTATCTTACATTGG	
espP2^C47A^-F/R	ATCCAAATCAGGCCTGGGGTGCTGTTGCAGGA	7,617
	CCAGGCCTGATTTGGATATTGTAAATCAGCAGT	
espP2^D144A^-F/R	ACGTTATGCCTCGGATGCCAAATTAGTCACTAAA	7,623
	CATCCGAGGCATAACGTTCCGTCCAGAATGGA	
espP2^H172A^-F/R	ACGCCACCGTAACCTTATGGGGAATTGAAGTT	7,618
	TAAGGTTACGGTGGCGTGCTGAGAAGTGAGTGCTGCA	
espP2^D194A^-F/R	GATGGATTAGTGCCTCTGTTAAGGATAAAGCTGGAAATCT	7,618
	CAGAGGCACTAATCCATCCTTTTTTTATCTTACC	
espP autotransporter domain-F/R	CGCGGATCCATATGGGCTAGAGTATTAGG	771
	CCGCTCGAGGAACGAGTATCTTACATTGG	

Restriction enzyme sites are underlined.

The mutants of EspP1 and EspP2 (EspP1 C47A, EspP1 D144A, EspP1 H172A, EspP1 D194A, EspP2 C47A, EspP2 D144A, EspP2 H172A, and EspP2 D195A) were generated by site-directed mutagenic PCR and confirmed by sequencing.

Purification of each His-tagged fusion protein was performed in *E. coli* BL21. Transformed *E. coli* was grown in 1 l LB medium plus 50 μg/ml kanamycin to an optical density at 600 nm (OD_600_) of 0.4–0.6 at 37°C and 200 rpm and induced with 0.8 mM isopropyl-β-D-thiogalactopyranoside (IPTG) at 37°C for 4 h. Bacterial cells were harvested, washed, resuspended in PBS and lysed by sonication. Following centrifugation at 13,000 × *g*, 4°C to clear the lysate, the His-tagged recombinant protein was purified through Ni Sepharose™ 6 Fast Flow (GE Healthcare Life Science, Pittsburgh, PA, United States).

### Extraction of culture supernatants and bacterial lysates

The protocol for extraction of the culture supernatants was performed as described previously (Orth et al., 2010) with slight modifications. *G. parasuis* strains CF7066, CF7066Δ*espP1*, CF7066Δ*espP2*, and CF7066Δ*espP1*Δ*espP2* were grown in 100 ml TSB overnight at 37°C.The culture supernatants collected *via* centrifugation and supernatants were passed through a 0.22-μm-pore-size filter. After adding 50% saturated ammonium sulfate into filtrates to precipitate the supernatants at 4°C, the precipitate was collected by centrifugation at 4°C and 12,000 × *g* for 30 min and the pellets were dissolved in PBS. Ammonium sulfate was removed during dialysis in PBS for 3 days.

After cultivation in TSB medium at 37°C for 12 h, bacterial culture was harvested, the cells were lysed by cell lysis buffer (Beyotime Biotechnology, Shanghai, China) without PMSF according to the manufacturer’s instruction.

### Immunoglobulin protease activity assay

*G. parasuis* strains were cultured to stationary phase in the presence of 2% heat-inactivated swine serum to investigate the immunoglobulin cleavage. Culture supernatants were collected and analyzed by Western blot with anti-IgA (Abcam, Cambridge, Cambridgeshire, Britain), anti-IgM (Invitrogen, Carlsbad, CA, United States) and anti-IgG (ABclonal, Wuhan, Hubei, China) antibodies.

To characterize the degradation specificity toward swine IgA, culture supernatants (15 μg) and bacterial lysates (15 μg) were incubated with swine IgA (3 μg) in PBS at 37°C for 0, 1, 2, 4, 6, and 8 h. Similarly, a time course consisting of 0, 1, 2, 4, 6, 8, and 10 min was performed in incubation of EspP1 (1 μg) and EspP2 (1 μg) with swine IgA (3 μg), and different amounts of recombinant protein (0.125, 0.25, 0.5, 1, 2, and 4 μg) were tested in 10 min. The reaction mixtures were separated by SDS-PAGE on 12.5% gels and blotted on PVDF membranes (Millipore, Billerica, MA, United States). After blocking membranes with 5% skim milk in TBST buffer at room temperature for 1 h, incubation with a 1:1,500 dilution of HRP-conjugated goat anti-pig IgA antibody. The detection of bound antibodies was accomplished using the ECL chemiluminescence kit (Vazyme Biotech, Nanjing, Jiangsu, China). The results of Western blot were analyzed by the software ImageJ ver. 1.53 (Bethesda, MD, United States). The relative protein level was calculated as follow: gray value of cleavage product/gray value of corresponding area of negative control. Each western blot analysis was repeated independently three times.

### Identification of cleavage site by N/C-terminal sequencing

IgA (10 μg) was incubated with recombinant EspP1 (5 μg) or EspP2 (5 μg), respectively, at 37°C for 8 h, separated by SDS-PAGE on 10% gel, stained with Coomassie brilliant blue R-250 (Sigma, Saint Louis, MO, United States). After destaining, the bands of cleavage products were cut out with a scalpel and subjected to N/C terminal sequencing, which based on LC–MS/MS, performed by Bio-Tech Pack (Beijing, China). Briefly, the sample was hydrolyzed by chymotrypsin and trypsin respectively, afterwards, LC–MS/MS was performed.

### Statistical analysis

Statistical analyses were performed using GraphPad Prism ver. 8.01 (San Diego, CA, United States). Results were compared by one-way ANOVA. A *p* value <0.05 was considered as significant.

## Results

### *Glaesserella parasuis* exhibits the capacity of cleaving swine IgA

**Figure 1:**
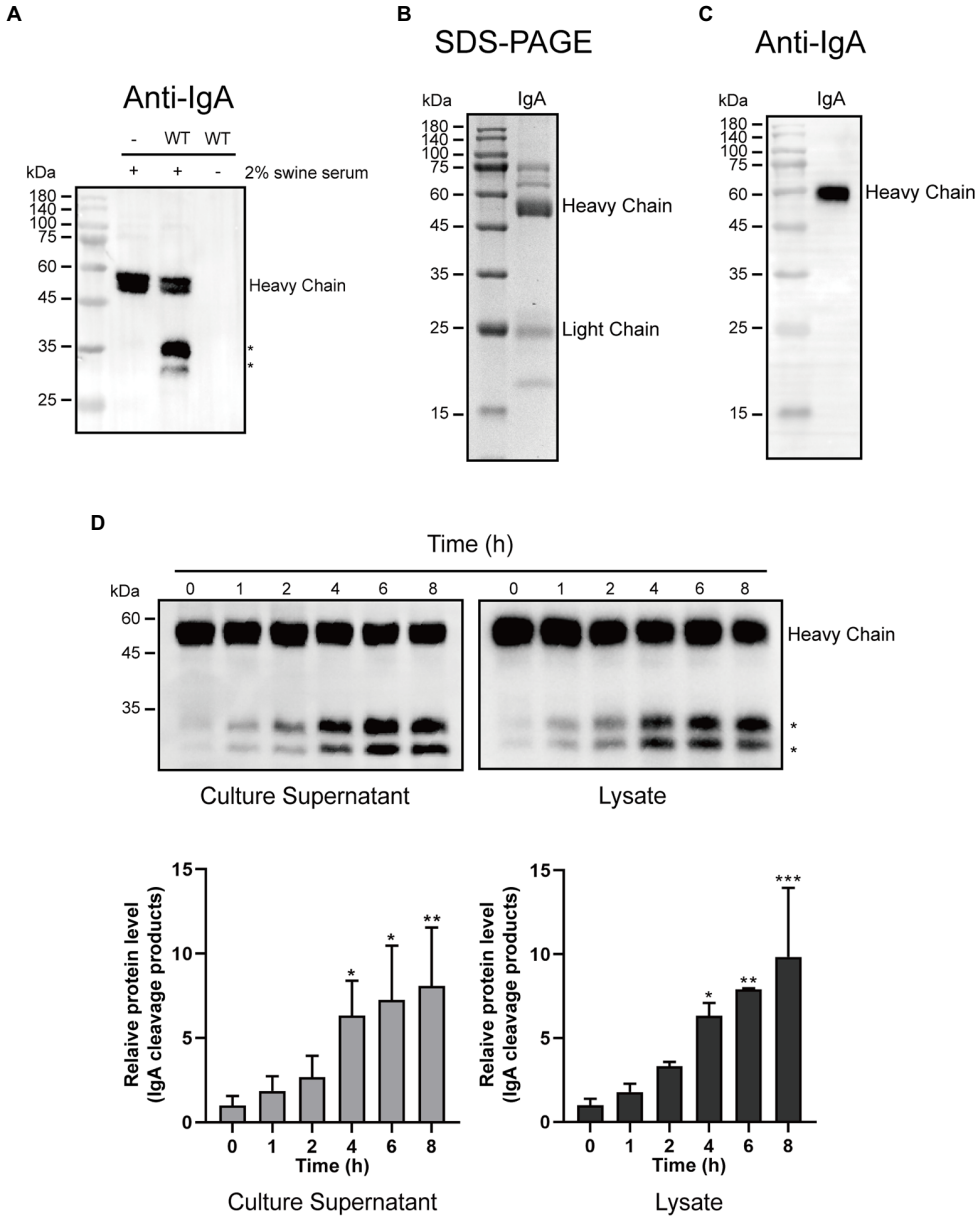
*Glaesserella parasuis* exhibits the ability to cleave swine IgA. **(A)** Culture supernatants of 2% heat-inactivated swine serum alone (−) and *G. parasuis* strain CF7066 cultured in the presence of 2% heat-inactivated swine serum to stationary phase were analysed by Western blot. IgA was detected with HRP conjugated Goat anti-Pig IgA antibody. The cleavage products are indicated with asterisk (*). SDS-PAGE **(B)** and Western blot **(C)** were used to assess the purification of swine IgA. **(D)** Culture supernatants and lysates were incubated with purified IgA at 37°C for different periods of time and analysed by Western blot.*p < 0.05, **p < 0.01, ***p < 0.001 using one-way ANOVA.

To investigate whether *G. parasuis* strain CF7066 possesses the capacity of cleaving swine IgA, the bacteria were cultured to stationary phase in the presence of 2% heat-inactivated swine serum. Cultures were spun down, and the supernatants were harvested and analyzed by Western blot. The cleavage products were only detected in the presence of both *G. parasuis* strain CF7066 and swine serum (Figure 1A). It suggests that *G. parasuis* strain CF7066 possesses the capability of degrading swine IgA. The swine IgA was then extracted from fresh swine colostrum *via* Sephadex G-200 and fiber gel DEAE-52. The integrity and purity of swine IgA was detected by SDS-PAGE and immunoblotting. Molecular weight of the heavy chain was consistent with theory (~60 kDa; Figures 1B,C).

Furthermore, we prepared culture supernatants and lysates from *G. parasuis* strain CF7066. Culture supernatants and lysates were incubated with purified IgA at 37°C for different periods of time (0, 1, 2, 4, 6, and 8 h), respectively. The cleavage products of approximately 27 kDa and 33 kDa were detected in a time-dependent manner (Figure 1D). It is plausible to postulate that the IgA protease in *G. parasuis* is a protease which can be secreted into the extracellular matrix. Collectively, these results suggest that *G. parasuis* exhibits the capacity of cleaving swine IgA.

### *Glaesserella parasuis* EspPs mediate cleavage of swine IgA heavy chain

**Figure 2:**
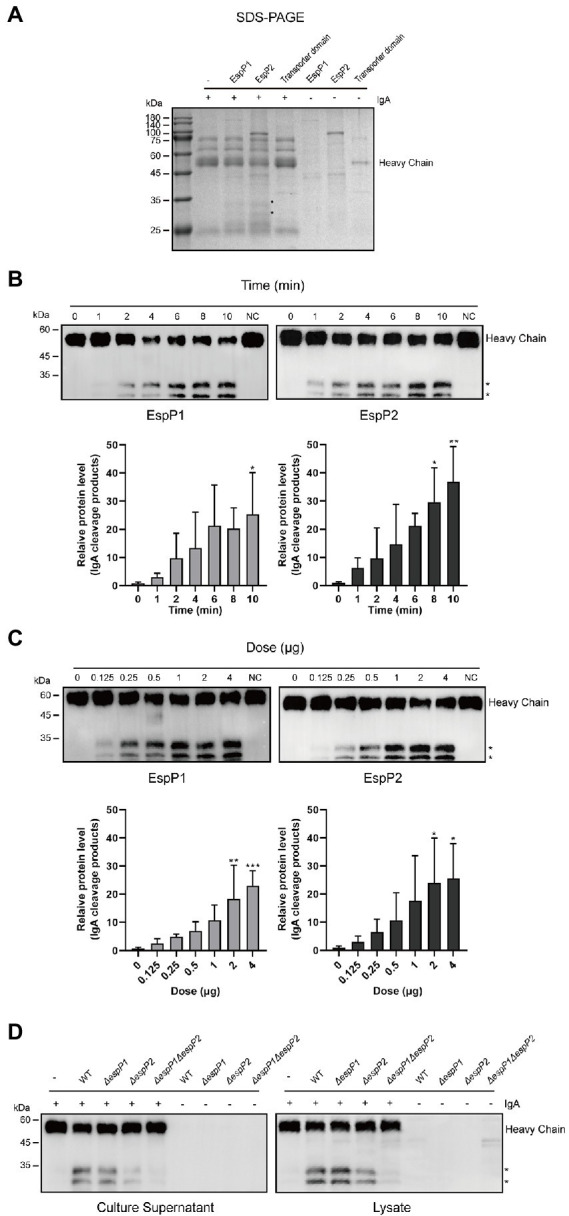
*Glaesserella parasuis* EspP1- and EspP2-mediated cleavage of swine IgA heavy chain. Cleavage of purified swine IgA by recombinant EspP1 and EspP2 was detected by SDS-PAGE **(A)** and Western blot **(B,C)**. The cleavage products are indicated with asterisk (*). And the IgA was cleaved in a time- **(B)** and dose- **(C)** dependent manner. **(D)** Lysates and culture supernatants of CF 7066 wild type (WT) and EspP1 and EspP2 defivient strain (AespP1, AespP2, and AespP1AespP2) were incubated with purified swine IgA, and the degradation of IgA was detected by Western blot. *p < 0.05, **p < 0.01, ***p < 0.001 using one-way ANOVA.

*Glaesserella parasuis* EspP1 and EspP2 are autotransporters which contain a Mac-1 domain within an immunoglobulin protease and an autotransporter domain. The autotransporter domain was also expressed and purified as a negative control. Recombinant proteins EspP1, EspP2 and the autotransporter domain were incubated with swine IgA at 37°C, respectively. As shown in Figures 2A–C we found that IgA was cleaved, respectively, by EspP1 and EspP2 in a time- and dose- dependent manner, and the cleavage products produced by EspP1 and EspP2 were detected at the same position as the culture supernatants, which indicated that EspP1 share the same cleavage sites with EspP2. Later, culture supernatants and lysates from EspP deficient mutants of *G. parasuis* strain CF7066 (CF7066Δ*espP1*, CF7066Δ*espP2* and CF7066Δ*espP1*Δ*espP2*) were collected to confirm whether IgA can only be cleaved by EspPs in *G. parasuis*. The result shows that both CF7066Δ*espP1* and CF7066Δ*espP2* still possess the capability to degrade swine IgA, however, the simultaneous deletion of *espP1* and *espP2* completely eliminate the IgA protease activity in *G. parasuis* (Figure 2D)*.* Together, these data further support the function of EspP1 and EspP2 in cleaving swine IgA.

### Protease activity of EspPs is affected by the putative active sites

**Figure 3:**
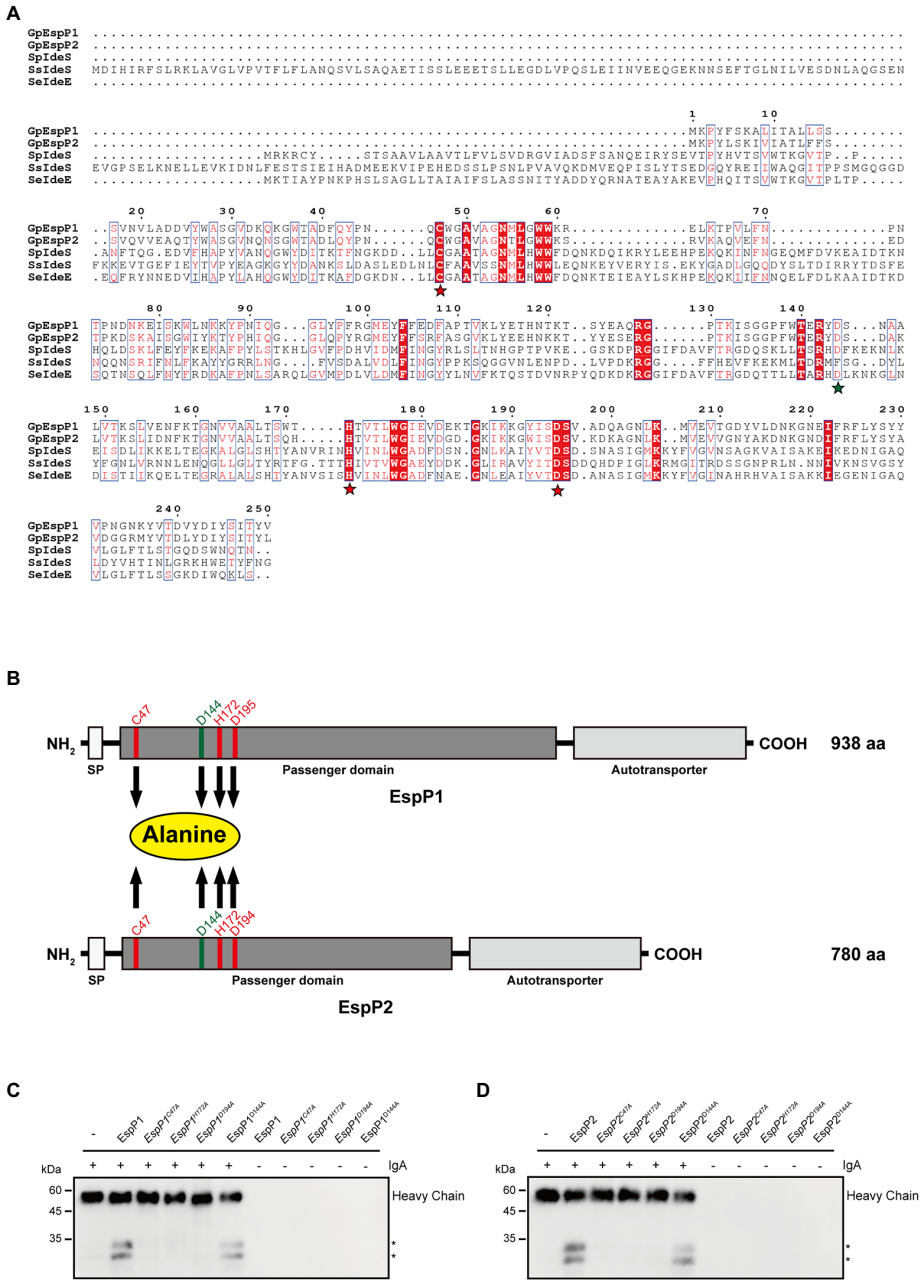
Protease activity of EspP1 and EspP2 are affected by the putative active sites which consisted of cysteine, histidine and aspartate. **(A)** The multiple-sequence alignment of *G. parasuis* EspP1 (aa 1-250) and EspP2 (aa 1-249) amino acids with the published sequence of *S. pyogenes* IdeS (aa 1-341), *S. suis* IdeS (aa 1-454) and *S. equi* IdeE (aa 1-349). The putative active sites were marked with yellow stars, and the D144 was acted as control, which was marked with a green star. **(B)** Schematic of EspP1 and EspP2. Positions of C47, H172, D194/D195, and D144 were highlighted and these amino acid residues were mutated to alanine separately. Protease activity of EspP1 **(C)** and EspP2 **(D)** disappeared when Cys-47, His-172, or Asp-194/Asp-195 were mutated to alanine (Ala). And the degradation activity of EspP1^D144A^ and EpspP2^D144A^ were not affected by the mutation.

Next, we moved on to explore whether the active sites have an effect on enzymatic activity. The active sites of Mac-1 consist of cysteine, histidine and aspartate, which are highly conserved. First, the multiple-sequence alignment of *G. parasuis* EspP1 and EspP2 amino acids with published sequence of *S. pyogenes* IdeS, *S. suis* IdeS and *Streptococcus equi* IdeE was performed. We found that Cys47, His172 and Asp194 of EspP1 corresponding to the active sites of *S. pyogenes* IdeS, Cys-94, His-262, and Asp-284 (Figure 3), which had been reported previously (Wenig et al., 2004; Agniswamy et al., 2006). It demonstrates that the putative active sites of EspP1 are C47, H172, and D194. Similarly, the putative active sites of EspP2 are C47, H172, and D195 (Figure 3). Later, the active sites of EspPs were mutated to alanine and the D144 was mutated to alanine as control. Following expression and purification of the EspP1 and EspP2 mutant proteins, the purified IgA (3 μg) was incubated with these mutant proteins (1 μg) respectively at 37°C for 10 min. As expected, cleavage of IgA by C47A, H172A, and D194A/D195A mutations of EspP1 and EspP2 cannot be detected through immunoblotting (Figures 3C,D). Nevertheless, the ability of EspP1_D144A_ and EspP2_D144A_ to degrade IgA did not disappear. It suggests that C47, H172, and D194/D195 are the active sites of EspP1 and EspP2, which has a negative effect on IgA protease activity.

### Swine IgA is cleaved within the Cα1 and Cα3 domains

**Figure 4:**
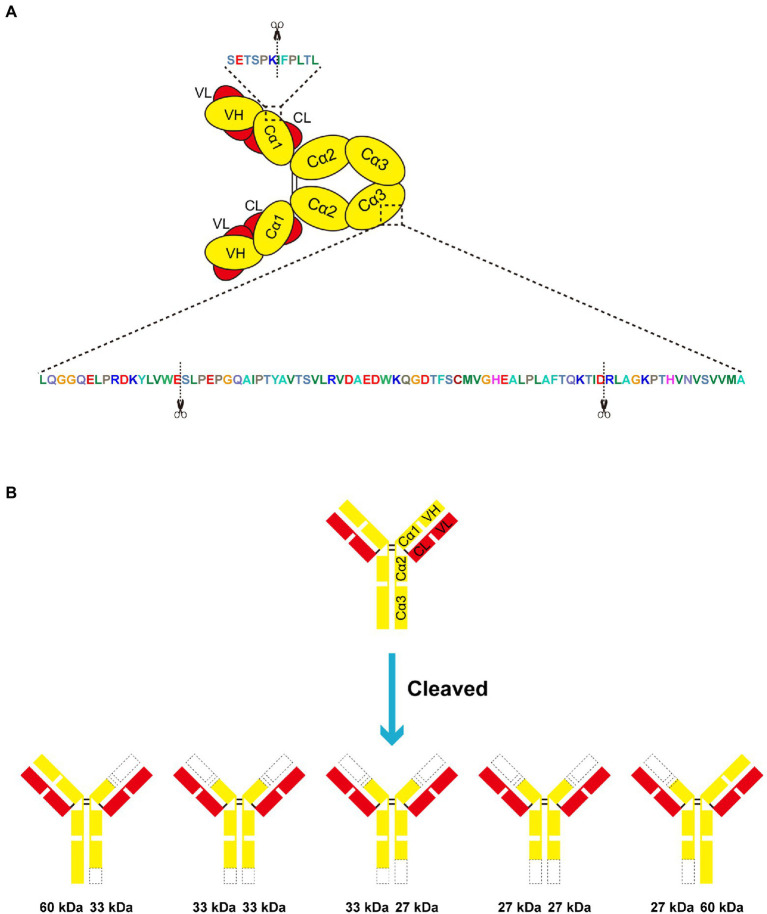
Swine lgA is cleaved within the Cd1 and Cu3 domain. **(A,B)** EspP1 and EspP2 have three different cleavage sites in swine IgA, which led to two different cleavage products with the molecular weight of ~33 and ~27 kDa under reducing condition. The cleavage sites are indicated with scissors.

Later, we sought to explore the cleavage site within swine IgA heavy chain. The bands of cleavage products were cut out and subjected to N/C terminal sequencing. Results of N/C terminal sequencing were shown in Supplementary Figure S1. These two degradation products share the same N terminal, the amino acid (aa) sequence IFPLTLGSS corresponding to the swine IgA Cα1 (Figure 4A). For ~33 kDa cleavage product, the C-termina sequence is LAFTQKTID, which corresponding to the Cα3 domain of swine IgA Fc region (Figure 4A). Similarly, the C-terminal sequence PRDKYLVWE of ~27 kDa is also within the Cα3 domain of swine IgA Fc region (Figure 4A). In conclusion, swine IgA is cleaved at three different positions to produce ~33 and ~27 kDa of cleavage products. There are five forms of cleaved IgA theoretically (Figure 4B), however, we can only observe the ~33 and ~27 kDa of cleavage products and ~60 kDa of full length under the reducing condition of SDS-PAGE.

### EspPs are IgA-specific proteases while *Glaesserella parasuis* exhibits IgM protease activity

**Figure 5:**
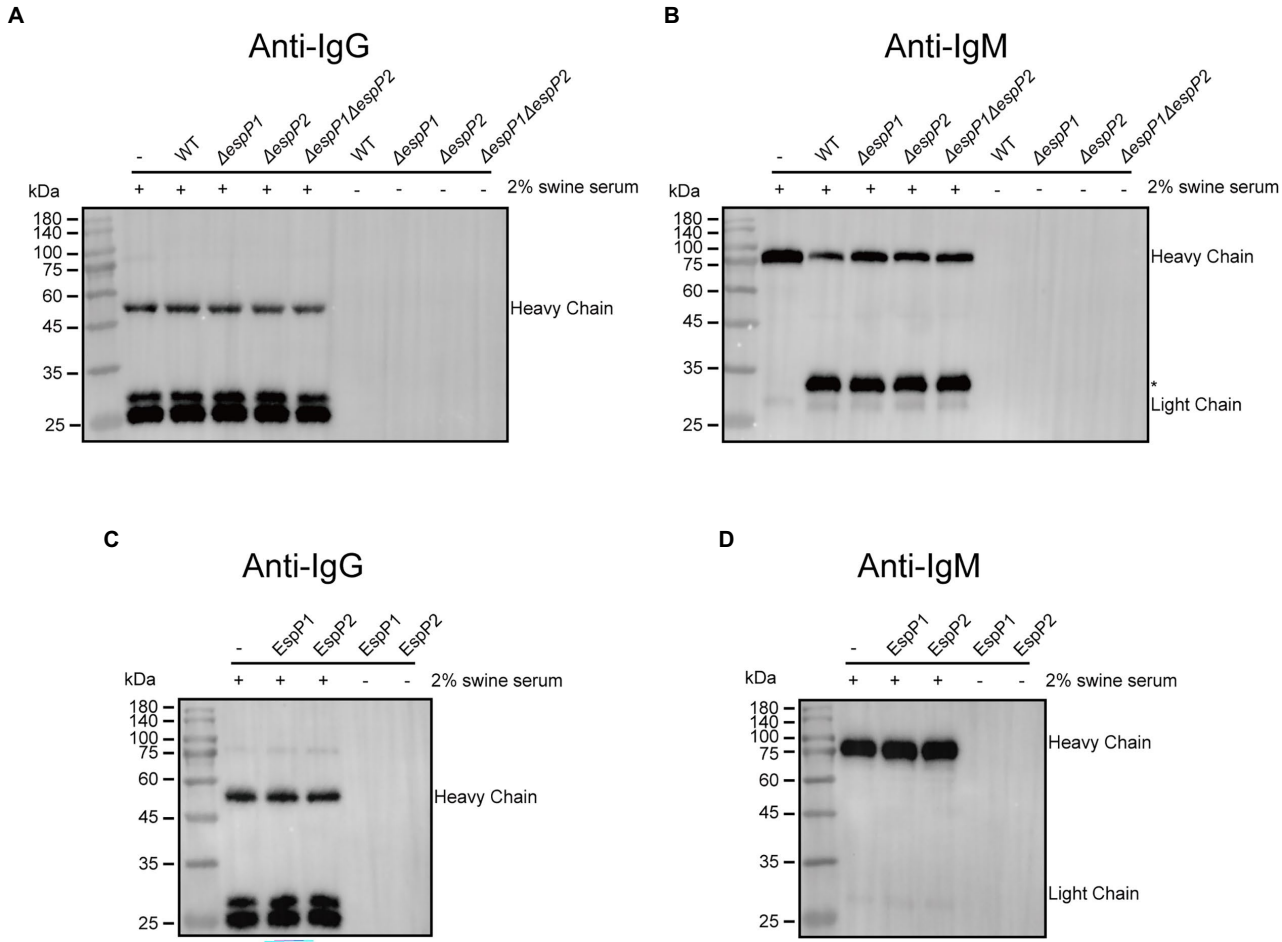
*Glaesserella parasuis* EspP1 and EspP2 are IgA-specific protease. **(A,B)** CF7066 and its derivatives EspPI and EspP2 deficient strains were incubated in the presence of 2% heat-inactiveted swine serum to stationary phase and detected by Western blot. The cleavage product is indicated with asterisk (*). **(C,D)** Recombinant EspP1 and EspP2 were incubated with 2% swine serum overnight at 37°C, and then subjected to Western blot.

To examine if *G. parasuis* possesses the degrading capacities to other immunoglobulins, strain CF7066 and EspP deficient mutants of CF7066 were cultured to stationary phase in the presence of 2% heat-inactivated swine serum. The culture supernatants were collected and analyzed by Western blot. As expected, degradation of IgG was not observed when CF7066 and EspP deficient mutants were cultured with swine serum (Figure 5A). Furthermore, degradation of IgM heavy chain was surprisingly observed and the degradation product of a ~ 33 kDa did not disappear when Δ*espP1*, Δ*espP2* or Δ*espP1*Δ*espP2* strains cultured with swine serum (Figure 5B). To verify if EspPs partake in the degradation of IgM, the incubation of recombinant EspPs and swine serum was carried out. And the result of Western blot shows that EspPs can degrade neither IgG nor IgM (Figures 5C,D). Thus, these results show that *G. parasuis* possesses the ability to degrade IgM unexpectedly. Nonetheless, EspPs are not involved in IgM cleavage, indicating they are IgA-specific proteases. It suggests that *G. parasuis* can secrete other proteases to cleave IgM which have never been reported.

## Discussion

In this study, *G. parasuis* EspP1 and EspP2 are identified as two novel IgA-specific proteases. The cleavage of IgA demonstrates that *G. parasuis* has the ability to thwart the host innate immune response. In addition to IgA cleavage, it is well established that *G. parasuis* possesses the ability of immune evasion, including phagocytosis resistance (Olvera et al., 2009; Costa-Hurtado et al., 2012), resistance against complement-mediated killing (Cerda-Cuellar and Aragon, 2008), and forming biofilm which protects the bacterium from antibody-mediated killing (Costerton et al., 1999; Jin et al., 2006). These strategies allow the microorganism to evade clearance of host immune system, and eventually, result in significant economic losses to the swine industry.

Interestingly, we found that *G. parasuis* IgA protease EspPs have the same catalytic triad residues with cysteine protease IdeS, which including cysteine, histidine and aspartate. EspP is known as a kind of extracellular serine protease (Pokharel et al., 2019). Serine protease EspP from *E. coli* contributes to biofilm formation (Xicohtencatl-Cortes et al., 2010), and it can cleave porcine pepsin A, coagulation V (Brunder et al., 1997) and complement factor C3/C3b (Orth et al., 2010). These cleavage activities are affected by the catalytic triad residues of serine protease consisting of serine, histidine and aspartate (Khan et al., 2011). However, there is no significant similarity between the passenger domain of *G. parasuis* EspPs and *E. coli* serine protease EspP. It demonstrates that *G. parasuis* EspPs play the function of IgA protease as a cysteine protease rather than a serine protease. As *espP* was identified as one of the potential virulence-associated genes that significantly upregulated *in vivo* (Mao et al., 2020), it indicates that *G. parasuis* IgA protease was up-regulated during infection and secretion of IgA-specific protease EspPs may serve *G. parasuis* to evade IgA-mediated mucosal immunity under physiological conditions. According to the results of N/C terminal sequencing, EspP1 and EspP2 have the same cleavage sites within swine IgA. However, the sequence alignment between passenger domain of EspP1 and EspP2 showed only 49% identity. And in terms of the amounts of amino acids, EspP1 has 159 more than EspP2. It demonstrates that, to some extent, there are still some differences between EspP1 and EspP2, it is possible that these differences manifest in their ability to exert other distinct functions, which have not been clearly explored yet. Hence IgA-specific proteases EspPs could consider as potential therapeutic candidates for *G. parasuis*. Neutralizing antibodies against EspPs could strengthen host mucosal immunity by avoiding destruction of mucosal anti-*G. parasuis* IgA by EspPs. The development of EspPs-based mucosal vaccine and small-molecule inhibitors of EspPs could be a new insight into prevention and control of *G. parasuis* in further study (Shehaj et al., 2019).

In the present study, we found that the heavy chain of swine IgA was cleaved in the Cα1 and Cα3 domains. IgA1 proteases produced by pathogenic bacteria such as *H. influenzae, S. pneumoniae*, and *M. haemolytica* are able to cleave in the hinge region of human IgA1 (Clementi et al., 2014; Janoff et al., 2014; Ayalew et al., 2019). IgA1 is cleaved in a specific site within hinge region, either a proline-serine or proline-threonine peptide bond (de Sousa-Pereira and Woof, 2019). Nonetheless, these specific sites are not present in swine IgA. The same as other immunoglobulins, both heavy chains (H) and light chains (L) of IgA are folded into variable (V) and constant (C) domains, which contains VH, Cα1, Cα2, Cα3, and VL, CL (de Sousa-Pereira and Woof, 2019). Fragment antigen-binding (Fab) region is constituted by VH, Cα1, VL, and CL, while Cα2 and Cα3 constitute the fragment crystallizable (Fc) region, and a flexible hinge region is present between Fab and Fc regions (Stanfield and Wilson, 2014). IgA is cleaved between Fab and Fc fragments which means Fab-mediated binding of antigen unable to link to Fc-mediated clearance mechanisms (Woof and Kerr, 2006). Unlike hinge region of human IgA1 which rich in proline, threonine and serine, hinge of swine IgA and human IgA2 are shorter than IgA1. The flexibility may impair while it would show less susceptibility to proteolysis. The finding of degradation of IgA by *G. parasuis* EspPs within Cα1 and Cα3 domains can interfere with the IgA-induced immune responses, as the antigen-binding region is parted from the Fc region. As the results of N/C terminal sequencing indicated that there is one cleavage site in Cα1 and two other cleavage sites in Cα3, it seems like that EspP1 and EspP2 have multiple proteolysis sites. Maybe the analysis of the structure of the interaction between EspPs and swine IgA would be helpful to better understand the molecular mechanism of interaction between EspPs and IgA. Interestingly, one of the cleavage sites also remain in ~33 kDa product. Therefore, theoretically, when there are enough EspPs and adequate incubation time, the ~33 kDa product would be re-cleaved to ~27 kDa, and there would be only ~27 kDa product left.

Next, we report for the first time that *G. parasuis* possesses the ability to degrade swine IgM, which is the first antibody secreted when exposure to exogenous antigens (Keyt et al., 2020). On the one hand, IgM defends against invasion of foreign microorganisms or mutated cells, such as cancer cells, through recognition and in conjunction with specific antigens on the surface of these threatens, this response involves engaging with macrophages, dendritic and mast cells (Vollmers and Brandlein, 2006; Keyt et al., 2020). On the other hand, the classical complement cascade initiated by IgM is also an effective method to target lysis of pathogens and cells. Following engagement of antigens, complement-mediated clearance is induced with a large conformational change which exposes the C1q binding motif on IgM (Sharp et al., 2019). There are only a few pathogens have been reported to cleave IgM so far. *S. pyogenes* SpeB and *Staphylococcus aureus* serine protease exhibit human IgM protease activity, and *S. suis* IdeS (also known as Mac-1) is a specific swine IgM protease (Prokesova et al., 1992; Collin and Olsen, 2001; Seele et al., 2013). In the present study, cleavage of swine IgM by *G. parasuis* was found and a ~ 33 kDa product was obtained. However, the Mac-1 containing autotransporters EspPs are not involved in this response. To localize the position of the IgM protease in bacterial cells, *G. parasuis* strain CF7066 and the culture supernatant and lysate of its derivatives were incubated in the presence of 2% heat-inactivated swine serum at 37°C for 12 h, respectively. The result of Western bot shows that the ~33 kDa product can only be observed when CF7066 was incubated with its lysate, while culture supernatant cannot (Supplementary Figure S2). It reveals that the IgM protease may locate in the outer membrane of *G. parasuis*. In the future, more efforts are needed to identify the IgM protease. And it is also necessary to investigate the effect of IgM cleavage on bacterial survival in swine blood and activation of the classical complement pathway.

In conclusion, our work determines a pair of novel and specific IgA proteases EspP1 and EspP2 expressed by *G. parasuis*. Swine IgA is cleaved by EspPs within Cα1 and Cα3 domains and EspPs function as cysteine proteases. Furthermore, *G. parasuis* possesses the capability to degrade swine IgM and this is reported for the first time. Identifying the underlying mechanisms of bacterial immune evasion may be able to shed new light on prevention and control of *G. parasuis*.

## Data availability statement

The original contributions presented in the study are included in the article/Supplementary material, further inquiries can be directed to the corresponding author.

## Author contributions

XC conceived and designed the research. ZW performed the experiments and analyzed the data. JG and KX performed the experiments. WZ, YL, and SW contributed reagents, materials, and analysis tools. ZW and XC wrote the manuscript. QH, XX, and XC contributed to funding acquisition and supervision. All authors contributed to the article and approved the submitted version.

## Funding

This work was supported by the China Agriculture Research System of MOF and MARA (number CARS-35).

## Conflict of interest

The authors declare that there are no financial or other relationships that might lead to a conflict of interest. All authors have seen and approved the manuscript.

## Publisher’s note

All claims expressed in this article are solely those of the authors and do not necessarily represent those of their affiliated organizations, or those of the publisher, the editors and the reviewers. Any product that may be evaluated in this article, or claim that may be made by its manufacturer, is not guaranteed or endorsed by the publisher.

## Footnotes

^1^ http://pfam.xfam.org/
