# Supplementary figures and images for: Glaesserella parasuis autotransporters EspP1 and EspP2 are novel IgA-specific proteases
Source: Front Microbiol. 2022 Dec 15;13:1041774. doi: 10.3389/fmicb.2022.1041774 (PMC9797811; doi:10.3389/fmicb.2022.1041774)

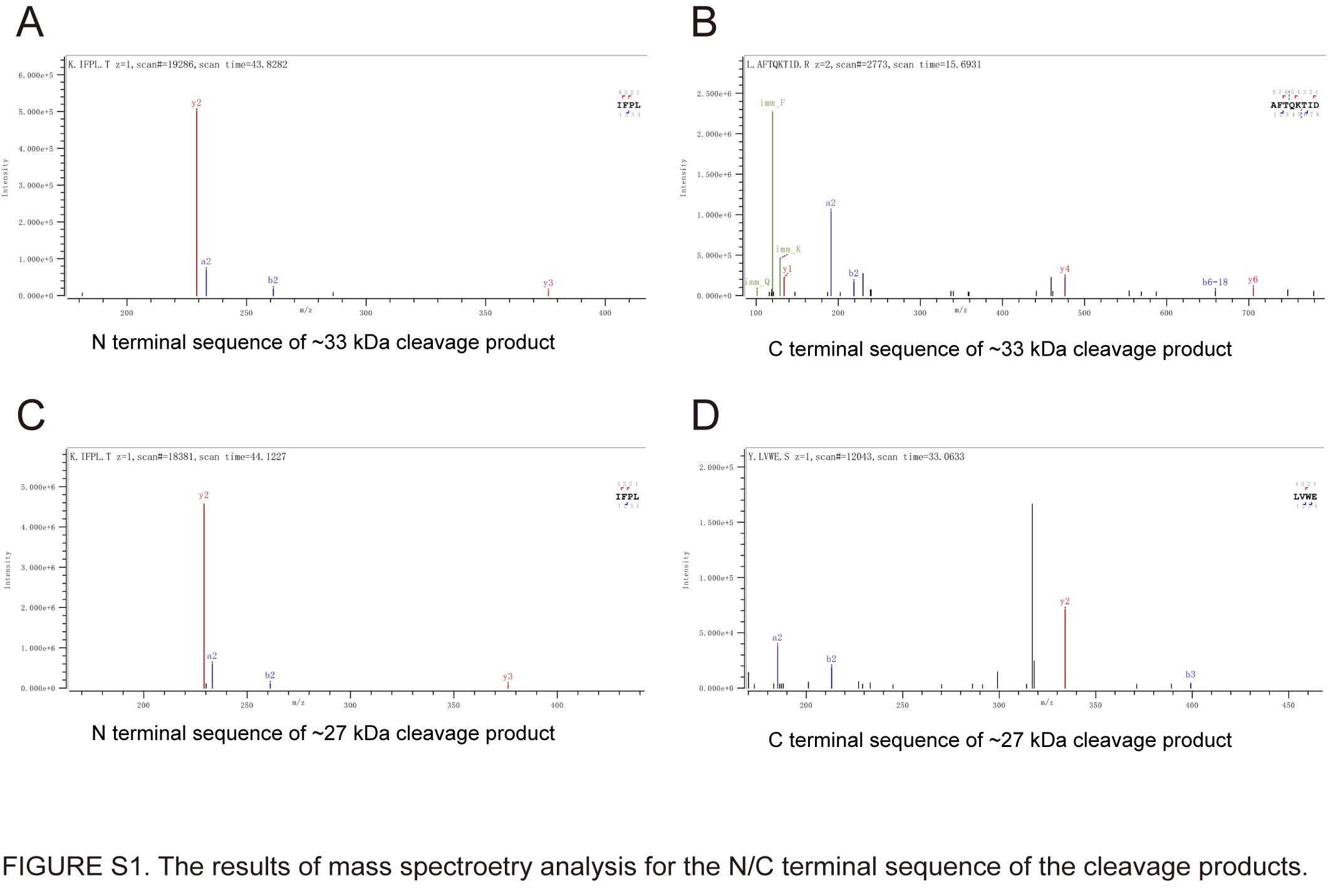

Supplement: Supplementary file 1 [file Image_1.TIF]

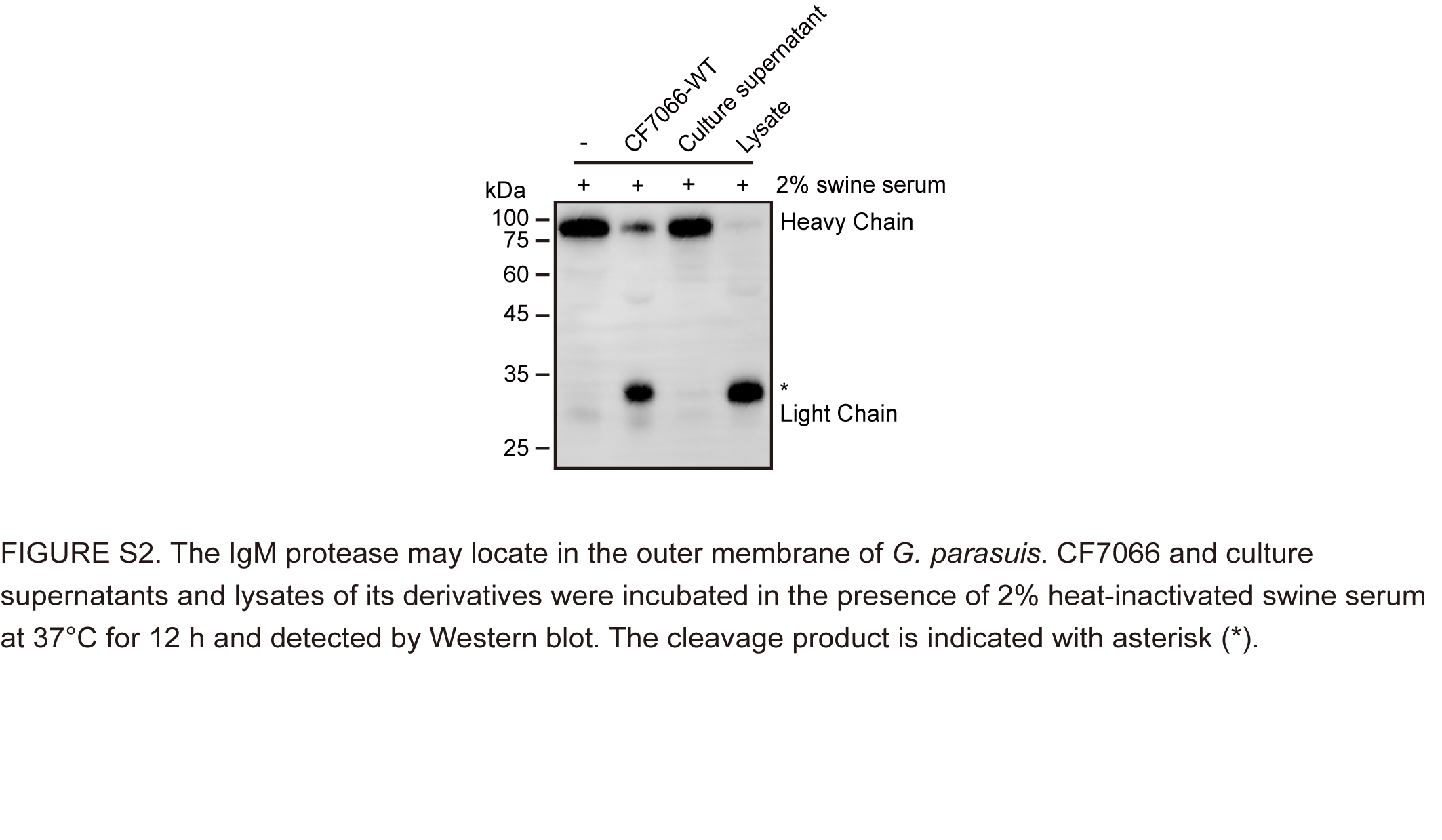

Supplement: Supplementary file 2 [file Image_2.TIF]
